# More than cost‐effectiveness? Applying a second‐stage filter to improve policy decision making

**DOI:** 10.1111/hex.13277

**Published:** 2021-06-01

**Authors:** Kaying Kan, Frederike Jörg, Joran Lokkerbol, Cathrine Mihalopoulos, Erik Buskens, Robert A. Schoevers, Talitha L. Feenstra

**Affiliations:** ^1^ Rob Giel Research Center Interdisciplinary Centre for Psychopathology and Emotion Regulation University Medical Center Groningen University Center for Psychiatry University of Groningen Groningen The Netherlands; ^2^ Research Department GGZ Friesland Leeuwarden The Netherlands; ^3^ Centre for Economic Evaluation and Machine Learning Trimbos Institute (Netherlands Institute of Mental Health and Addiction) Utrecht The Netherlands; ^4^ Deakin Health Economics School of Health and Social Development Faculty of Health Deakin University Geelong Australia; ^5^ Department of Epidemiology University Medical Center Groningen University of Groningen Groningen The Netherlands; ^6^ Faculty of Economics and Business University of Groningen Groningen The Netherlands; ^7^ Interdisciplinary Centre for Psychopathology and Emotion Regulation University Medical Center Groningen University Center for Psychiatry University of Groningen Groningen The Netherlands; ^8^ Department of Science and Engineering Groningen Research Institute of Pharmacy University of Groningen Groningen The Netherlands; ^9^ National Institute for Public Health and the Environment (RIVM) Bilthoven The Netherlands

**Keywords:** cost‐effectiveness analysis, decision making, health technology assessment, major depressive disorder, patient participation, priority setting

## Abstract

**Background:**

Apart from cost‐effectiveness, considerations like equity and acceptability may affect health‐care priority setting. Preferably, priority setting combines evidence evaluation with an appraisal procedure, to elicit and weigh these considerations.

**Objective:**

To demonstrate a structured approach for eliciting and evaluating a broad range of assessment criteria, including key stakeholders’ values, aiming to support decision makers in priority setting.

**Methods:**

For a set of cost‐effective substitute interventions for depression care, the appraisal criteria were adopted from the Australian Assessing Cost‐Effectiveness initiative. All substitute interventions were assessed in an appraisal, using focus group discussions and semi‐structured interviews conducted among key stakeholders.

**Results:**

Appraisal of the substitute cost‐effective interventions yielded an overview of considerations and an overall recommendation for decision makers. Two out of the thirteen pairs were deemed acceptable and realistic, that is investment in therapist‐guided and Internet‐based cognitive behavioural therapy instead of cognitive behavioural therapy in mild depression, and investment in combination therapy rather than individual psychotherapy in severe depression. In the remaining substitution pairs, substantive issues affected acceptability. The key issues identified were as follows: workforce capacity, lack of stakeholder support and the need for change in clinicians’ attitude.

**Conclusions:**

Systematic identification of stakeholders’ considerations allows decision makers to prioritize among cost‐effective policy options. Moreover, this approach entails an explicit and transparent priority‐setting procedure and provides insights into the intended and unintended consequences of using a certain health technology.

**Patient contribution:**

Patients were involved in the conduct of the study for instance, by sharing their values regarding considerations relevant for priority setting.

## 1 INTRODUCTION

Countries have limited public resources to invest in health care. Technological innovations and resource constraints continuously challenge health‐care priority setting.^1^ Indeed, maintaining comprehensive, high‐quality, sustainable and affordable health‐care packages entails difficult choices. Accordingly, numerous frameworks have been proposed to guide health‐care decision making.^2^, ^3^, ^4^, ^5^, ^6^

Most developed countries currently apply formal health technology assessments (HTAs), notably for pharmaceuticals, to substantiate reimbursement decisions. Generally, HTAs follow distinct phases: problem definition during a scoping phase, an evaluation according to different assessment criteria during an assessment phase and an appraisal of all available information by an independent multidisciplinary committee to provide policy recommendations during an appraisal phase.^7^, ^8^

This organizational structure reflects growing recognition that the evaluation of evidence and public engagement techniques should be incorporated into priority‐setting approaches.^9^ HTA can be strengthened by a systematic approach to include robust evidence about patients’ perspectives and by ensuring effective engagement of patients in the entire HTA/appraisal process to create a fair deliberative process.^10^ Several forms of public or patient engagement (e.g. telephone surveys, questionnaires or public meetings) can occur at various levels of the HTA process.^11^ For example in the Netherlands, the assessment criteria are set and followed by stakeholder consultation rounds, where medical experts, patients, professional associations or other relevant health‐care stakeholders may be consulted during the appraisal. In the UK, the National Institute for Health and Care Excellence (NICE) can consult a citizen council to elicit public perspectives on overarching moral and ethical issues that NICE should consider when providing guidance (e.g. societal values to be considered in decisions about trade‐offs between equity and efficiency).^12^

The degree of public engagement (deliberative or non‐deliberative participation) differs by country,^13^ and the extent to which citizens’ or patients’ inputs influence the final decision remains unclear.^11^, ^14^ Involving experts such as clinicians and health providers may help to provide important insights into various domains and the context in which technologies are used. However, to improve patient outcomes, and to take into consideration the needs of the group that is affected most by decisions regarding health technologies, requires the involvement of patients. Therefore, a deliberative decision‐making process, in which experts and clinicians as well as patients are systematically involved, may be the way forward.^11^, ^13^

Apart from the regulatory framework, guiding principles or criteria applied within this framework are critical. A World Health Organization survey found that the majority of member states reported using a formal HTA process to inform coverage decisions.^14^ The main criteria applied in HTA were safety, clinical effectiveness and economic and budgetary considerations. Acceptability to patients and health‐care organizations, equity and ethical issues, and feasibility considerations rarely receive systematic attention.^14^ Moreover, the findings of HTA‐performing organization(s) are considered advisory rather than mandatory for policy decisions. A comparison of criteria applied within international HTA frameworks reveals that some criteria are perceived to be important across systems, but there is no consensus on a universal set of core criteria to inform priority setting.^15^

The importance of incorporating a broad range of criteria other than effectiveness and cost‐effectiveness in the decision‐making process is twofold. First, it is rare that all consequences and costs can be included within technical cost‐effectiveness calculations (e.g. informal caregiver impacts are often not considered or valued). Furthermore, there are ‘due‐process’ considerations in decision making that do not feature explicitly in estimations of incremental cost‐effectiveness ratios. For example, parenting interventions designed to prevent anxiety disorders in children appear to provide good value for money.^16^ However, several key stakeholders have highlighted issues pertaining to this intervention, including community concerns associated with the stigmatization of positively screened preschool children and parents’ reluctance to participate in such interventions.^16^

To date, while many studies recommend incorporating additional criteria and perspectives in health‐care priority setting, few studies have actually demonstrated how such an approach could be implemented. Importantly, methods for weighing the opinions of different stakeholders, incorporating these opinions and deciding what criteria to use remain unclear.^17^ Thus, the aim of this study was to demonstrate a structured methodology for eliciting and weighing additional criteria jointly with the results of cost‐effectiveness analyses by systematically involving diverse stakeholders in a different way than having a deliberative commission. We used the principles guiding the ‘second‐stage filter’ approach, derived from the Assessing Cost‐Effectiveness (ACE) priority‐setting approach,^18^ and built onto a set of cost‐effectiveness analyses regarding depression treatment, undertaken by Lokkerbol et al^19^ in the Netherlands. We began our qualitative analysis of important criteria, other than cost‐effectiveness, with an assessment of the core criteria commonly used in existing ACE studies.^20^, ^21^ Subsequently, we investigated other considerations relevant to the local Dutch mental health‐care context for inclusion in the ‘second‐stage filter analysis’. We have adopted a systematic approach using focus group discussions (FGDs) and semi‐structured interviews to elicit information from key stakeholders: health‐care professionals, patients, and health provider and health insurer representatives.

## 2 METHODS

### 2.1 Study design

We used a case study in depression care to illustrate the application of a structured appraisal methodology using preselected criteria and eliciting additional criteria. Qualitative research techniques were used with key stakeholders to elicit and evaluate criteria other than cost‐effectiveness for a list of potential cost‐effective substitute interventions.

### 2.2 Cost‐effective substitute interventions for depression care

Lokkerbol et al developed a health economic substitution algorithm to identify pairs of treatment interventions from among those currently in use that could result in a more cost‐effective health‐care system for patients with major depressive disorders.^19^ Intervention pairs were identified via health‐care substitution, by (partly) investing in a more cost‐effective intervention and simultaneously disinvesting in a less cost‐effective intervention. The cost savings from disinvestment would cover the investment in more cost‐effective interventions. Comparable economic evaluation approaches that jointly consider investment and disinvestment decisions are called the ‘step in the right direction approach’, and is also used in programme budgeting and marginal analysis.^22^, ^23^ We applied the economic analysis described by Lokkerbol et al,^19^ using regional estimates for the incidence and prevalence of depression in Friesland, a province in the Northern Netherlands. Estimates were based on NEMESIS‐2,^24^ and we used updated intervention costs in accordance with the latest Standard of Care for depressive disorders.^25^

### 2.3 Selection of appraisal criteria

The ACE priority‐setting approach was developed in Australia and has been used extensively to support health‐care policy in areas not covered by formal HTA. This structured approach is aimed at reducing methodological inconsistencies across economic evaluations. It explicitly considers both formal cost‐effectiveness analyses for reducing methodological confounding and ‘due‐process’ decision‐making considerations, largely obtained via a Steering Committee of stakeholders. Legitimacy is achieved through explicit discussions of other criteria important to decision making—commonly referred to as the ‘second‐stage filter’ criteria.

Previous ACE studies have identified the following key criteria essential for decision making: equity and ethical issues, acceptability to key stakeholders, strength of evidence, feasibility considerations, and other important beneficial or harmful effects not captured in the technical analysis.^18^ Importantly, these criteria can change according to the requirements of each decision‐making context. We used these second‐stage filter criteria in our study as a starting point to elicit information from key stakeholders for the list of substitute interventions derived from the model‐based cost‐effectiveness analysis.^19^

### 2.4 Elicitation approach

FGDs and semi‐structured interviews were conducted to elicit information on criteria from key stakeholders. While focusing on the above‐mentioned criteria, the interview guide also allowed for the inclusion of other relevant issues (for details, see Supplementary Material A). We used a phenomenological approach to obtain stakeholders’ views and values on important priority‐setting considerations in depression care. This approach elicits individuals’ experiences of a certain phenomenon.^26^ It simultaneously attempts to set aside preconceived assumptions about experiences of a particular situation.

### 2.5 Participants

Key stakeholder participants were patients with depression or a history of depression, health‐care professionals who treat depression, the director of the largest regional specialist mental health‐care organization and a medical advisor of the health insurer with the largest market share (approximately 61%) in the region.

FGDs with patients stimulated exchanges of knowledge and experiences and their perceptions regarding potential substitute interventions. We conducted semi‐structured interviews with the remaining stakeholders. Because of time constraints and logistical issues, five of the ten health‐care professionals preferred to fill in a questionnaire covering the same topics.

Patients and health‐care professionals were selected through purposive sampling. Patients were eligible to participate in this study if they currently or previously suffered a depressive disorder and underwent several treatments; were willing to provide informed consent; and agreed with the collection and use of audio recorded anonymized data. Health‐care professionals were eligible for participation if they had experience in treating patients with depressive disorder and were willing to share their perceptions regarding the substitution pairs. Full details regarding the recruitment of participants are described in Supplementary Material B.

The largest specialist mental health‐care organization and health insurer were selected through convenience sampling. The health insurer perspective was represented by the medical advisor in mental health. The director of the mental health‐care organization represented the provider perspective. All participants were contacted by telephone or email for an eligibility check. Three patients dropped out of the study due to personal circumstances (a broken toe, a funeral and a hospital admission, respectively) and one interview with a clinician was unusable due to faulty audio equipment.

### 2.6 Data collection and analysis

Data collection took place between December 2017 and August 2018. All interviews were audio recorded and transcribed verbatim. Field notes were taken during the FGDs and semi‐structured interviews.

Data were analysed using ATLAS.ti version 8.2, a qualitative analysis software. One researcher coded the data (K.K.), and two researchers (F.J. and T.F.) participated in peer‐debriefing to improve the credibility and validity of the results. Thematic content analysis was performed on the data using the one‐sheet‐of‐paper method.^27^ Patients and health‐care professionals provided feedback on the findings to verify the accuracy of the interpretations of the transcripts. Themes were derived from the data but were mostly driven by the interview guide topics.

We constructed a summary table based on our analysis of the interview data. Subsequently, conclusions on issues identified for each criterion, displayed by coloured cells, were converted into appropriate recommendations on each substituted intervention pair for decision makers. Three researchers independently judged the importance of the identified issues. The researchers discussed their differences, eventually reaching consensus. Quotes by different stakeholders were used to illustrate the findings.

## 3 RESULTS

### 3.1 Cost‐effectiveness analyses

Table 1 presents the intervention substitution pairs using the health economic substitution algorithm. Substitution of an intervention by an alternative can potentially improve cost‐effectiveness within the mental health‐care system. A detailed description of non‐standard interventions is given in Supplementary Material C.

**TABLE 1:** Cost‐effective substitute interventions for depression care

No.	Treatment for	Investment in	Disinvestment in
1	Mild depression	therapist‐guided Internet‐based cognitive behavioural treatment	individual cognitive behavioural therapy
2	Moderate depression	therapist‐guided Internet‐based cognitive behavioural treatment	individual cognitive behavioural therapy
3	Moderate depression	pharmacotherapy (3‐6 months)	individual cognitive behavioural therapy
4	Severe depression	antidepressant medication (12 months)	individual psychotherapy (8‐24 sessions)
5	Severe depression	antidepressant medication (12 months)	combination therapy
6	Severe depression	antidepressant medication +General Practice assistant^†^	individual psychotherapy (8‐24 sessions)
7	Severe depression	antidepressant medication +General Practice assistant^†^	antidepressant medication (12 months)
8	Severe depression	antidepressant medication +General Practice assistant^†^	combination therapy
9	Severe depression	combination therapy	individual psychotherapy (8‐24 sessions)
10	Prevention: Recurrent depression	mindfulness‐based cognitive behavioural therapy	clinical management with maintenance medication (12 months)
11	Prevention: Recurrent depression	mindfulness‐based cognitive behavioural therapy	preventive cognitive (behavioural) therapy
12	Prevention: Recurrent depression	interpersonal psychotherapy	clinical management with maintenance medication (12 months)
13	Prevention: Recurrent depression	preventive cognitive (behavioural) therapy	clinical management with maintenance medication (12 months)

^†^ Hypothetical scenario: effectiveness of antidepressant medication with increased adherence rate caused by guidance of General Practice assistant.

### 3.2 Qualitative data analysis

Data were analysed for 23 participants: 12 patients, 9 clinicians, 1 director of a mental health‐care organization and 1 medical advisor from the health insurer. The average durations of the FGDs (three in total) and semi‐structured interviews were 85 and 45 minutes, respectively. We established data saturation, defined as the point when no new information or themes were observed in the data, in May 2018 and June 2018 after conducting three FGDs and ten interviews, respectively.

We first elaborate on two specific examples (substitution pairs 1 and 2 in Table 1). Next, we present the salient findings, including judgements, for each of the substitution pairs.

#### 3.2.1 Illustration of two examples

Table 2 presents a summary of the findings for substitution pairs 1 and 2: investment in therapist‐guided Internet‐based cognitive behavioural treatment (iCBT) and disinvestment in individual cognitive behavioural therapy (CBT) for (1) mild and (2) moderate depression. Issues identified for each criterion are described in more detail below, and the findings are illustrated with respondents’ quotations (Table 3). Similar tables for all remaining substitution pairs (3‐13) are provided in Supplementary Material D.

**TABLE 2:** Priority‐setting considerations based on different stakeholders’ perspectives on investment in therapist‐guided, Internet‐based cognitive behavioural treatment (iCBT) and disinvestment in individual cognitive behavioural therapy (CBT) for mild and moderate depression

Level of evidence	‐ Clinicians doubt treatment effects in routine practice; not all clinicians have experience in providing Internet‐based treatments. Clinicians with experience in providing Internet‐based treatments have no doubts ‐ Evidence of treatment effectiveness is required for mild and moderate depression and for primary care services versus specialized care services
Equity and equality considerations	Potential causes of increased inequalities/inequities are: ‐ An ageing population, (computer) illiteracy, low socio‐economic status of patients, patients with sight and hearing deficiencies, psychiatric comorbidities, and intellectual disabilities ‐ Treatment effects are more obvious in younger patients. However, for adolescents, in particular, connecting with others could be important in a digital world ‐ Patients are not open to this form of therapy ‐ Treatment is more suitable for patients with mild depression, but its suitability for patients with moderate depression is questionable because of their decreased treatment adherence and difficulties putting things into perspective Potential causes of decreased inequalities/inequities are: ‐ Patients’ insights ‐ Individual clinicians’ increased caseloads and reductions in waiting lists ‐ Less travel time and costs ‐ Insights of patients who experience barriers to therapy sessions and/or patients who have difficulty expressing themselves ‐ Treatment occurs in patients’ own environments, and they have opportunities to re‐read the information at leisure
Feasibility of implementation	Positive: ‐ In organizations that already provide this form of treatment, clinicians are qualified, and no issues are anticipated ‐ Implementation is less intensive for clinicians; whereas a CBT session requires an hour, a therapist‐guided Internet‐based cognitive behavioural treatment requires 30 minutes Neutral: ‐ More efficient deployment of staff is required ‐ The recommendation is to start with a pilot initiative to gain some experience Issues: ‐ Training of qualified personnel is required regarding to e‐health guidance and a different form of contact ‐ The application should be interactive, user‐friendly and catchy ‐ Mental health‐care procurement is dependent on the resources of care organizations ‐ Training is required when intervention is provided as a component of primary care services
Acceptability to stakeholders	Positive: ‐ Acceptable in its blended form in combination with face‐to‐face therapy ‐ Could be more effective: working method is more task‐oriented ‐ Good experiences ‐ Structured moments of contact via the Internet, during which clinicians can provide supportive and stimulating feedback Neutral: ‐ Patients’ preferences remain important ‐ Suitable for moderate depression along a continuum (if not too severe); CBT treatment is appropriate for more severe cases Issues: ‐ The extent of clinicians’ openness and willingness to adapt to this method of interacting with patients ‐ Clinicians’ handling of patients’ self‐responsibility ‐ Suitability and quality of the e‐health material (adaptation for different target populations, content tailored for specific patients, and based on CBT principles) and ease of use for both clinicians and patients ‐ Challenges of convincing patients and clinicians to accept the innovative intervention ‐ Requires patients’ trust in clinicians (as a precondition) ‐ Requires patients to work independently during treatment, demonstrating self‐discipline and responsibility ‐ Not suitable or questionable suitability for patients with moderate depression. Suggestions: begin with medication and then continue with therapist‐guided Internet‐based cognitive behavioural treatment. Requires patient stability and structure in daily routine. Not suitable for patients with suicidal thoughts. More intensive initial treatment, more frequent sessions, or in conjunction with medication ‐ CBT remains important for treating moderate depression ‐ Clinicians’ unfamiliarity with this type of treatment ‐ Possible resistance from the older generation of clinicians; doubts about treatment effectiveness
Other effects not captured in modelling	Positive: ‐ Patient empowerment ‐ Current waiting lists for CBT are long, and depression can start to resolve (prevention of deterioration) ‐ Antidepressant prescriptions or CBT treatments may be reduced or avoided Negative: ‐ Non‐verbal communication and interaction is missing in the e‐health component; patients’ openness to this form of therapy is unclear ‐ Requires a therapeutic relationship prior to implementing the e‐health component of the intervention ‐ Physically attending the treatment motivates patients to stay active, and maintains structure in daily routine

**TABLE 3:** Illustrative quotes for each criterion

Criterion	Quotations for illustration
Level of evidence	Quote 1: 'Good proposal, you are less caught up in the hassle of every day, so to say. You are really putting in a lot more work. I have had good experiences, but I have also noticed, the main practitioners, ie the psychiatrists, clinical psychologists, psychotherapists, they often forget that we have that option. So, it is a matter of repeating it during meetings, also consider that option. Because it simply fades away again, people have not yet gotten it into their system.' (Participant 23, woman, age 35)
Equity and equality considerations	Quote 2: 'Maybe you don't reach the entire target group, but that doesn't happen for CBT either. I don't think you should let that stop you because in every form of therapy, you will have people who are making progress and people who are not benefitting, and then you should pay more attention to them [the latter].' (Participant 20, man, age 35)
Feasibility of implementation	Quote 3: ‘People always think more e‐Health means less staff’. And I ask myself … that is what I am wondering. Because you also need to properly support eHealth. And yes, you still need the face‐to‐face contacts anyway. So, the story is about a more efficient use of staff.‘ (Participant 15, man, age 56)
Acceptability to stakeholders	Quote 4: ‘As a clinician, you obviously loose some of the control, you are going to hand more control to the patient. That I really think is a very good development, but for a clinician it may be a bit uncomfortable. I think you need to gain more experience, learning to confide in that system.’ (Participant 20, man, age 35) Quote 5: ’So acceptance of the clinicians’ side. Them actually taking that step, stepping away from the comfort zone of the treatment room, and switching to a different kind of making contact with the. patients. Uh, it's the suitability of the material, right? So, of the e‐health modules that we have.’ (Participant 15, man, age 56) Quote 6: ’You miss the non‐verbal element of course; but it's just another way of making contact, but I notice that the therapists themselves seem to find it pleasant.’ (Participant 23, woman, age 35)
Other effects not captured in modelling	Quote 7: ’And you allow, moreover, in the entire system of the empowering of the patient, you give them the tools to do things. Tools that you can use after the treatment has ended.’ (Participant 22, man, age 45‐55)

##### Level of evidence

Most of the health‐care professionals who were unfamiliar with iCBT believed that it could be meaningful for patients who are not severely depressed. They indicated that it should be at least as effective as regular face‐to‐face CBT. Health‐care professionals already familiar with iCBT reported seeing good outcomes and did not have any doubts about the effectiveness in clinical practice (Table 3, quote 1). One health‐care professional remarked that some of her colleagues were more reluctant to apply iCBT because they lacked information regarding its effectiveness in clinical practice. The representatives of the mental health‐care organization and health insurer felt that iCBT was worth investing in, and both stakeholders endorsed provision of this intervention and evaluations of its effects on patients with *mild* depression. By contrast, several stakeholders were reluctant to offer therapist‐guided iCBT to patients with *moderate* depression because they anticipated limited treatment adherence within this group.

##### Equity and equality

All the stakeholders indicated that the substitute intervention would be justified for the majority of patients with *mild* depression. They all agreed that a minority of patients with mild depression might not prefer this type of intervention for reasons such as (computer) illiteracy or low socio‐economic status (Table 3, quote 2). Most stakeholders considered iCBT to be more appropriate for younger patients, as most of them are familiar with digital devices. One patient did not recommend it for adolescents, especially those who needed social connections. Conversely, others believed that iCBT provides a solution for young people who feel ashamed about undergoing psychological treatment. It could reduce no‐show in patients who require therapy but whose time is occupied by work/school. Moreover, it could overcome barriers for a certain group of patients, as some patients find it difficult to tell their stories face‐to‐face.

Some patients and health‐care professionals argued that iCBT would not be equivalent to existing therapies for *moderately* depressed patients because some patients find structure in daily routine, discipline, stability and independent functioning challenging. In such cases, expectations of motivating oneself to participate in an Internet‐based treatment may not be realistic.

Other important equity and equality considerations included provision of treatment in patients’ own environments, which saves travel time and costs. The treatment also elicits patients’ insights and the possibility of re‐reading the information in patients’ own time and pace. Finally, it may increase the productivity of health‐care professionals, reducing waiting lists.

##### Feasibility of implementation

None of the stakeholders expected major implementation issues for patients with either *mild* or *moderate* depression. Some health‐care professionals and the director of the mental health‐care provider expected that iCBT would require more efficient staff deployment (Table 3, quote 3).

The stakeholders also did not anticipate major implementation issues with an Internet‐based application that is user‐friendly and well integrated within current systems. In organizations yet to offer iCBT and in primary care, proper training of qualified personnel regarding different ways of interacting with patients (e.g. less non‐verbal communication in iCBT) is required. The health insurer's medical advisor was very open to iCBT procurement whether mental health‐care organizations could provide such interventions.

##### Acceptability

Stakeholders prioritized investments in iCBT over individual CBT for *mild* depression. One clinician stated that task‐oriented interventions like iCBT could even be more effective than CBT for some patients, as treatments are structured and entail regular online contact. In addition, patients regularly receive supportive and stimulating feedback, while non‐verbal information can still be conveyed through face‐to‐face contact. Clinicians experienced in using iCBT have rarely encountered resistance from patients.

Stakeholders mentioned some potential risks for iCBT, which would require patients to be self‐disciplined. Moreover, face‐to‐face contact remains important for maintaining the therapeutic relationship. Patients’ acceptance is needed, so the suitability of the intervention requires verification.

Some other challenges were mentioned by health‐care professionals and representatives of the health insurer and mental health‐care provider: health‐care professionals should be open and willing to adapt to a new form of contact with patients, and trust patients to be self‐responsible (Table 3, quotes 4, 5 and 6). Furthermore, patients’ preferences for treatment types remain important and stakeholders agreed that health‐care professionals cannot be expected to adopt this treatment easily and immediately. Finally, they mentioned that the e‐Health material should be appropriate for the target population, and ease of use for both health‐care professionals and patients was considered a necessary condition.

Most stakeholders questioned the suitability of iCBT for treating *moderate* depression, because of patients’ decreased concentration and moods. For iCBT to work, patients need a stable environment, structure in daily routine, and they should not be severely depressed. Some stakeholders suggested beginning with a more intensive intervention until the depression stabilizes and subsequently providing iCBT or providing it along with medication. Several stakeholders mentioned that conventional face‐to‐face CBT remains important in treating moderate depression.

##### Other relevant non‐efficiency considerations

Both patients and health‐care professionals felt that patients’ trust in therapy and the therapeutic relationship were crucial for good treatment outcomes. For patients without a social network or structured routine, travelling to treatment centres could have benefits, as it promotes some scheduled activities. However, providing more iCBT could lead to a reduced need for antidepressant prescriptions and CBT treatment, thereby saving costs and shortening waiting lists. Finally, patients and health‐care professionals felt that iCBT could increase patient involvement in therapy, leading to patients regaining control over themselves more rapidly, with expected consequences for recovery times (Table 3, quote 7).

In sum, for treating *mild* depression, investment in therapist‐guided iCBT and disinvestment in individual CBT seems to be a promising potential substitute intervention pair with no major issues anticipated. However, for treating *moderate* depression some concerns remain.

#### 3.2.2 Assessment of all substitution pairs incorporating all criteria

Table 4 presents the conclusions for all the assessed substitution pairs incorporating all considerations. Green, orange and red cells denote no, possible and considerable issues/concerns, respectively, from the stakeholders’ perspectives. The last column depicts policy considerations for each substitution pair.

**TABLE 4:**
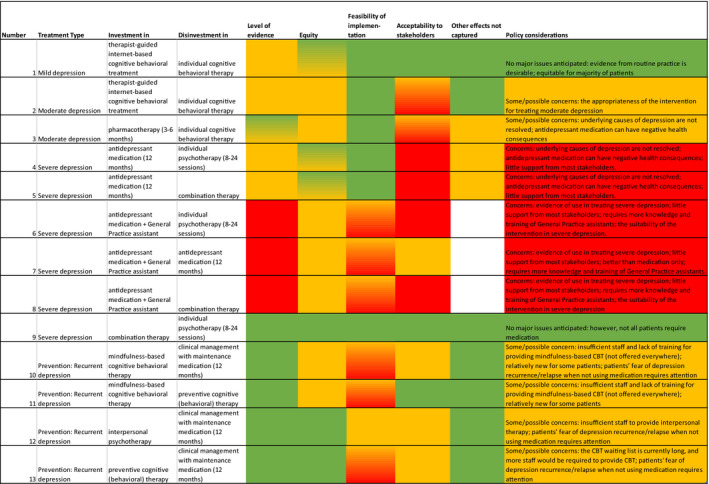
Summary of the results of the second‐stage filter analysis

Key issues regarding the level of evidence criterion were related to clinical effectiveness as opposed to efficacy, and the outcomes for specific target groups. In addition, health‐care professionals noted that patients’ preferences, which they wished to consider, could sometimes conflict with treatment protocols and guidelines, which were perceived as being rigid. The stakeholders also pointed to equity and equality issues. For example, stakeholders found it inequitable that some people cannot benefit from certain treatment for instance because of computer illiteracy. They also mentioned that several substitute interventions that targeted patients with severe depression were seen as interventions that were too ‘light’ for the type of depression concerned. For example, as treatment with medication only without psychological treatment does not provide patients any insights or personal behavioural changes stakeholders perceived it as an inequality issue for this specific group of patients. This substitute intervention was found to be more suitable for patients with milder complaints. They preferred more intensive treatment over under‐treatment. The main feasibility issue identified was insufficient staff to implement the interventions, for example when investing in mindfulness‐based cognitive behavioural therapy for patients with recurrent depressions. For several substitute interventions (eg the interventions with support from a General Practice assistant), training of staff is required to increase feasibility of implementation. The main acceptability issues stemmed from the clinicians’ work culture, including reluctance to pass on patients to their colleagues, who might be better equipped to provide necessary interventions, or to transfer responsibilities to their patients.

Finally, independent of a specific substitute intervention, personalized treatment and shared decision making remain essential, and were prioritized over and above economic considerations by all of the stakeholders. Further, stakeholders noted that patients’ social networks or contexts could significantly affect treatment outcomes. Economic evaluations do not usually cover this aspect. From the perspectives of the mental health‐care provider and health insurer, the long‐term cost‐effectiveness of interventions and prevention of future recurrences were deemed important.

## 4 DISCUSSION

The aim of this study was to demonstrate how the structured approach may add to the deliberative commission approach in that viewpoints on a range of pre‐specified domains are elicited from diverse stakeholders, while also leaving room for other domains. Next to the perspectives of experts, the insights and experiences of patients provide an additional perspective into the intended and unintended consequences of using a certain health technology. A large number of patients and other relevant stakeholders can potentially be involved, and their viewpoints processed to provide an overview of perspectives to increase the extent to which their inputs influence final decisions. By understanding the views and values of diverse stakeholders, balanced against the requirements of fair health‐care resource distribution, decision makers are facilitated in well‐informed health‐care priority‐setting decisions. The study also demonstrated that incorporating such criteria was possible in an alternative procedure than the ACE‐setting, using systematic qualitative research methods.

We found that for the majority of substitute interventions that could improve the cost‐effectiveness of depression care, stakeholders addressed one or more concerns. Six substitute interventions showed possible issues/concerns, and five substitute interventions showed considerable issues/concerns. Tackling anticipated issues prior to implementing proposed substitute interventions increases the chances of successful implementation and the likelihood of eliciting key stakeholders’ support. From the stakeholders’ perspectives, two substitute interventions remained promising after considering the cost‐effectiveness criterion as well as all other relevant criteria.

In the particular case study discussed here, investment in therapist‐guided iCBT and disinvestment in individual CBT appears to be a promising strategy for patients with mild depression. Health‐care professionals and patients expressed concerns regarding its suitability for patients with moderate depression due to an anticipated limited treatment adherence. Evidence suggests however that the adherence to therapist‐guided iCBT appears to be adequate, equals the adherence to face‐to‐face CBT^28^ and can meet the same outcomes as face‐to‐face CBT, also in moderate depression.^29^, ^30^, ^31^ After further investigation of the quality and validity of the expressed concerns, any remaining obstacles can be taken away by, for example, informing or educating specific stakeholders. These concerns, biased or not, are however important to take into account when informing HTA recommendations, as they influence acceptability and feasibility of implementation. Interestingly, the 2019‐2020 COVID‐19 pandemic has compelled clinicians to provide patients with online therapy. Moreover, patients have had to participate in online therapy during the pandemic because of the temporary unavailability of regular face‐to‐face contact. From clinicians’ and patients’ perspectives, online therapy, video calling and chatting appear to offer a possible solution when conventional CBT is not an option.^32^, ^33^ This finding sheds new light on the relative importance of the identified considerations and provides an opportunity to evaluate the outcomes of widespread use of online therapies using real‐world data in the future.

Using qualitative methodology, we elicited key stakeholders’ perspectives on a wide range of criteria. Other methods, such as sending out questionnaires, do not allow stakeholders to elaborate freely, and important considerations may therefore be missed. Recent policies have not captured all of the criteria considered for priority setting.^34^, ^35^, ^36^ In the Netherlands, the formal assessment criteria applied are necessity, evidence of effectiveness, cost‐effectiveness and feasibility.^37^ Our findings suggest that it could be worthwhile to incorporate additional criteria like acceptability or equity during the appraisal phase by eliciting stakeholder views on these issues.

Although this methodology's implementation requires additional resources and time, we found that its execution was highly feasible. Participants were willing to share their views and values regarding the substitution pairs. Because stakeholders’ views and values may conflict, all concerned stakeholders should be represented to enable informed decision making. Apart from carefully selecting criteria for priority setting, we recommend leaving room for other criteria not considered during the interviews, so that crucial issues are not missed.

In our study, the researchers made an overall judgement considering all of the identified issues per substitution pair to demonstrate which substitute interventions could potentially contribute to a cost‐effective health‐care system. As recommended by Facey et al, professionals experienced in research should be responsible for gathering evidence and its presentation and interpretation in HTA. More intense collaboration between the HTA community and researchers is needed.^10^ We recommend that existing national appraisal committees in which all stakeholders are represented should be tasked with the final evaluation process, to ensure a more standardized process.

Our study has several limitations. First, not all stakeholders are equally well represented in terms of numbers. However, we included different stakeholders’ perspectives in our analysis to capture the breadth of opinions. In addition, data saturation was reached as the stakeholders independently raised similar issues.

Second, various criteria, defined a priori, were evaluated during the interviews, along with other criteria that the stakeholders considered relevant. However, the stakeholders did not rank the relative importance of the criteria. Nevertheless, the FGDs revealed that patients believed that cost should not be the foremost consideration. For health‐care professionals, experience through clinical practice sometimes prevails over research evidence. Further, a shortage of personnel to administer the treatment was considered an important bottleneck. This finding indicates that different stakeholders prioritize different criteria, although practical or logistic impediments were considered to be objective constraints, rather than stakeholders’ opinions or preferences.

Finally, although patients were knowledgeable about depression treatments, most patients were not well versed in health economic rationing. To ensure that patients were adequately informed, we explained the rationale of the study, and economic rationing in general, to them prior to conducting the FGD.

### 4.1 Implications for policy and future research

Recent trends like value‐based health care, but also the long‐standing tradition of HTA stress that cost‐effectiveness is not the only relevant aspect for priority‐setting decisions. Current policy processes address these broader perspectives mostly in the appraisal phase, using deliberative panels. The current study piloted an alternative approach that adds to deliberative panels by offering a structured approach resulting in an overview of stakeholders’ views and that has the potential to increase the extent to which their inputs influence final decisions.

Because stakeholders' values relating to allocation of care could differ, the process of mapping all of their issues and concerns could contribute to a transparent methodology of informed decision making. Accordingly, future studies should investigate whether the systematic provision of information relating to other criteria alongside the efficiency criterion results in improved decision making. Our findings contribute to the growing body of evidence that demonstrates that patients can contribute to public argumentation and may determine the acceptability of proposed decisions.^10^, ^38^, ^39^ Understanding patients’ values and the impact of a specific technology/intervention on patients’ lives and their visions and requirements concerning the intervention are highly relevant as patients are most directly affected by the technology. It will be most relevant for reimbursement policy concerning treatments that currently lack formal inclusion of cost‐effectiveness, for example in the area of mental health care or prevention.

This systematic methodology can strengthen the HTA process by ensuring the disclosure of key stakeholders' values and preferences. A deliberative process in which diverse stakeholders are included helps to contextualize technologies and enables informed decision making. It highlights interventions that can be easily and quickly rolled out, as well as those that require further consideration before roll‐out can be considered. How the findings of current deliberation will inform the final decision‐making process and their impacts on service delivery is not yet clear. However, a major health insurer and a mental health‐care provider indicated their intention to take the results presented into account in the coming contract negotiations for multi‐year agreements starting from 2022. The application of this methodology requires additional resources and time compared with a cost‐effectiveness evaluation alone. However, when smooth implementation follows, this investment may prove worthwhile.

## 5 CONCLUSIONS

Improving the health‐care system through interventions that meet the efficiency criterion may have limitations because they do not include additional considerations relevant to priority setting. Access to diverse stakeholders’ views and values regarding these criteria, via a systematic embedded qualitative approach, enables decision makers to make better‐informed decisions and appropriate judgments when setting health‐care priorities. It also informs decision makers on issues that could impede successful adoption of the intervention, allowing them to tackle these issues. Furthermore, addressing such criteria in an open and explicit manner increases the transparency of the priority‐setting process.

## CONFLICT OF INTEREST

All authors have no conflict of interest to declare.

## AUTHORS' CONTRIBUTIONS

Kan, Jörg, Feenstra, Lokkerbol and Mihalopoulos conceptualized and designed the study. Kan, Lokkerbol, Feenstra and Jörg analysed the data. All authors interpreted the data. Kan, Jörg, Feenstra and Mihalopoulos drafted the manuscript. All authors revised the manuscript and finally approved the version to be published.

## Supporting information

Supporting information A

Supporting information B

Supporting information C

Supporting information D

## Data Availability

We are legally and ethically not allowed to publicly post our data set. Participants provided informed consent for the data collection and the use of audio recorded anonymized data for this particular study only.
