# Supplementary material for: More than cost‐effectiveness? Applying a second‐stage filter to improve policy decision making
Source: Health Expect. 2021 Jun 1;24(4):1413–23. doi: 10.1111/hex.13277 (PMC8369110; doi:10.1111/hex.13277)
Supplement: Supplementary file 1 — Supporting information A [file HEX-24-1413-s001.docx]

**Supplemental Material A – Interview guide topics**

| Topics |  |
| --- | --- |
| Introduction | Familiarity with the different interventions  General opinion on the use of economic considerations for priority setting |
| Acceptability / desirability | General opinion on the acceptability of investment and disinvestment of the substitution pair. Pro’s and con’s  Desirable changes needed for acceptability |
| Feasibility of implementation | Consequences for general practice/ mental healthcare procurement / mental healthcare organization  Changes needed to increase feasibility of implementation |
| Equity considerations | Suitability substitution pair for entire target population  Consequences substitution pair for equity and equality |
| Level of evidence | Importance of level of evidence  Experiences in practice with regard to effectiveness |
| Other considerations | Other considerations to take into account |
