# Supplementary material for: More than cost‐effectiveness? Applying a second‐stage filter to improve policy decision making
Source: Health Expect. 2021 Jun 1;24(4):1413–23. doi: 10.1111/hex.13277 (PMC8369110; doi:10.1111/hex.13277)
Supplement: Supplementary file 2 — Supporting information B [file HEX-24-1413-s003.docx]

**Supplemental Material B - Details of the recruitment of patients and healthcare professionals and the conduct of the focus group interviews and semi-structured interviews in the study**

In line with the Dutch Medical Research involving Human Subjects Act, the Medical Ethics Review Board (METc) of the University Medical Center Groningen exempted this research from full review. We obtained a waiver from the METc because there was not an infringement of the physical and/or psychological integrity of the study participants. Prior to the start of the focus groups, written informed consent was obtained from all participants. All participants agreed on audiotaping the interviews and usage for scientific research after anonymization.

Patients were recruited in several mental healthcare organization in the north of the Netherlands connected to the Rob Giel Research center (a collaboration of regional mental healthcare organizations in the northern part of the Netherlands). Mental healthcare organizations provide inpatient, outpatient, and community care treatment to patients with (severe) mental health problems. Client Councils of these mental healthcare organizations distributed the call via flyers and a call was posted on the website of the University Medical Center Groningen (https://www.umcg.nl/NL/UMCG/Afdelingen/Universitair_Centrum_Psychiatrie/Nieuws/Paginas/oproep-improve-studie.aspx).

Similarly, clinicians from various mental healthcare organizations in the northern part of the Netherlands were recruited and interviewed. In the Netherlands, every citizen is mandatory insured with standard health insurance. Citizens have an option to take out additional insurance to cover services not included in the standard package.

Focus group interviews took place in meeting rooms of a mental healthcare organization. Healthcare professionals were interviewed in their consultation room, the member of the Executive Board was interviewed in the mental healthcare organization and the medical advisor of the health insurer was interviewed at the headquarters of the health insurance company. Besides the participants and the researchers, nobody else was present during the conduct of the interviews. After the first interview, we made small adaptations in the interview guide. All participants participated once, and no repeated interviews were carried out.
