# Supplementary material for: More than cost‐effectiveness? Applying a second‐stage filter to improve policy decision making
Source: Health Expect. 2021 Jun 1;24(4):1413–23. doi: 10.1111/hex.13277 (PMC8369110; doi:10.1111/hex.13277)
Supplement: Supplementary file 3 — Supporting information C [file HEX-24-1413-s002.docx]

**Supplemental Material C – Description of interventions that may not be frequently used internationally**

| **Intervention** | **Description of the intervention** |
| --- | --- |
| Therapist-guided internet-based cognitive behavioral treatment (iCBT)^1,2^ | iCBT is a web-based, manualized, therapist-assisted cognitive behavior therapy. The therapy consists of patients performing homework assignments that implement common CBT interventions. Therapist guidance consist of feedback and instructions that are tailored by the therapist to the patient’s situation using motivational techniques. Treatment duration varies from 5-16 weeks, in which feedback is provided by the therapist two times a week. |
| Antidepressant medication + General Practice assistant* | In the Netherlands, the mental health system is divided into General Practitioner care including General Practice assistants specialized in mental health, generalist mental healthcare and specialist mental healthcare. Currently, patients with minor psychiatric complaints receive treatment at the GP with assistance from specialized GP assistants.  The intervention means that patients receive antidepressant medication and that patients are supported by the specialized GP assistants. |

**^1^** Ruwaard J, Lange A, Schrieken B, Emmelkamp P. Efficacy and effectiveness of online cognitive behavioral treatment: A decade of interapy research. Stud Health Technol Inform. 2011;167:9-14.

^2^ Ruwaard J, Broeksteeg J, Schrieken B, Emmelkamp P, Lange A (2010) Web-based therapist-assisted cognitive behavioral treatment of panic symptoms: A randomized controlled trial with a three-year follow-up. J Anxiety Disord 24: 387–396.
