# Supplementary material for: More than cost‐effectiveness? Applying a second‐stage filter to improve policy decision making
Source: Health Expect. 2021 Jun 1;24(4):1413–23. doi: 10.1111/hex.13277 (PMC8369110; doi:10.1111/hex.13277)
Supplement: Supplementary file 4 — Supporting information D [file HEX-24-1413-s004.docx]

**Supplemental Material D – Findings of the remaining substitution pairs 3-13**

**Intervention substitution pair 3: Stakeholders’ perspectives on investment in pharmacotherapy (3-6 months) and disinvestment in individual cognitive behavioral therapy for moderate depression**

| Level of evidence | - Questionable long-term effectiveness |
| --- | --- |
| Equity and equality considerations | Potential causes of increased inequalities/inequities are:   - Religion; some religions do not allow the use of medication - Medication only may result in under-treatment, and no insights and/or, personal (behavioral) changes are achieved   Potential causes of decreased inequalities/inequities are not mentioned |
| Feasibility of implementation | Positive:   - No major issues anticipated, it is also current practice   Issues:   - More psychiatrists needed for prescribing antidepressants - Higher workload for psychiatric nursing specialists and psychiatrists |
| Acceptability to stakeholders | Positive:   - Some patients respond well to medication only - Acceptable if it is the patients’ preference or if patients are not willing to undergo psychological treatment - Desirable in vital depressions   Issues:   - Not all patients need medication for a moderate depression - Medication only alleviates depressive symptoms, it does not solve the depression completely - Medication is supportive, in (behavioral) psychotherapy patients learn to deal with underlying/predisposing/persistent factors and patients obtain tools to be able to cope. This would be lacking if investing in pharmacotherapy only - Good and affordable investment on short-term but individual psychotherapy is needed once the depression is more stable - Dependent on the origin/ symptomatology of the depression; in the case of factors within a person, the person must also learn skills by psychological treatment, otherwise there is an increased risk of relapse - Patients’ fear for side effects (which can be decreased with psycho-education) |
| Other effects not captured in modelling | Positive:   - Makes patients more open to other forms of therapy   Negative:   - Undesirable addictive effect with negative health consequences - Patients feel like not being themselves, their thinking and reasoning is flattened - Metabolic adverse events; rapid aging, hypertension |

**Intervention substitution pair 4 and 5: Stakeholders’ perspectives on investment in antidepressant medication 12 months and disinvestment in individual cognitive behavioral therapy /** **combination therapy for severe depression**

| Level of evidence | - Long-term effectiveness of antidepressant medication for 12 months should be well proven - Long-term effects are important |
| --- | --- |
| Equity and equality considerations | Potential causes of increased inequalities/inequities are:   - Results in under-treatment, psychological treatment is needed - Religion; some religions do not allow the use of medication - Patients not willing to take medication   Potential causes of decreased inequalities/inequities are:   - Patients with difficulties in cognitive thinking due to their depression |
| Feasibility of implementation | Positive:   - No major issues anticipated, it is also current practice   Issues:   - Proper alignment needed and a clear plan of action - The availability of psychiatrist or other personnel allowed to provide the pharmacological intervention. - Higher workload for psychiatric nursing specialists and psychiatrists - Psychiatrists need to see the patient more frequently, and thereby less able to see more new patients in a day |
| Acceptability to stakeholders | Positive:   - Medication provides a boost and makes patients ready to go into therapy in the beginning, thereafter gradually decrease the amount of medication - Effective in case of suicidal thoughts - In minor cases this could work - Not desirable to withhold patients from medication - This is the recommended approach for a vital depression - in severe depression antidepressants are desirable   Issues:   - Medication in severe depression is only supportive, it is not desirable to provide medication only - Medication only alleviates symptoms, makes a depression milder and bearable, psychotherapy, structure in daily routine is crucial - Coaching and support is still needed to work on structure in daily routine and to resume daily activities - Medication does not solve the (cause of) depression, the underlying factors should be tackled. It remains important to obtain tools to cope with depression - Psychiatrists need to see the patient more frequently; uncomplicated depression without psychiatric comorbidities in severe depression is rare - In non-vital depression CBT treatment will be more effective - Combination therapy is common practice, and is the preference of most patients |
| Other effects not captured in modelling | Positive:   - Antidepressant use make patients ready to start other forms of therapies   Negative:   - Medication tackles sadness but therapy addresses how to deal with sadness - Addiction to some antidepressant medication - Undesirable addictive effect with negative health consequences - Probability of recurrence - Patients feel like not being themselves, their thinking and reasoning is flattened - (Metabolic) adverse events; rapid aging, hypertension - Long term effects after no use of medication |

**Intervention substitution pair 6, 7 and 8: Stakeholders’ perspectives on investment in antidepressant medication 12 months + General Practice assistant and disinvestment in individual psychotherapy /** **antidepressant medication 12 months / combination therapy for severe depression**

| Level of evidence | - Questionable effects of a General Practice assistant in severe depression - Evidence of effectiveness of a General Practice assistant in severe depression is important |
| --- | --- |
| Equity and equality considerations | Potential causes of increased inequalities/inequities are:   - Burden for General Practice assistants - Severely depressed patients deserve more knowledgeable and experienced staff - Under-treatment   Neutral:   - Investment in antidepressant medication 12 months + General Practice assistant does not necessarily lead to inequalities   Potential causes of decreased inequalities/inequities are:   - Shorter waiting lists in specialized mental healthcare organization - Decreased workload in specialized mental healthcare organization, General Practice assistants have more time |
| Feasibility of implementation | Positive:   - Additional investment in experts by experience might be beneficial - Less provision of specialized mental healthcare   Neutral:   - Need to consider the consequences, patients still get coaching and support and personal attention   Issues:   - Training of General Practice assistants are necessary - General Practice assistants’ knowledge is questionable for severe cases - Knowledge of General Practice assistant is currently lacking in most cases, they are not all specialized in mental health, training is needed or psychiatrist in the General Practice office can be a solution - General Practice assistants are not qualified to prescribe medication - If antidepressants are provided by a psychiatrist, communication between psychiatrist and General Practice assistant is difficult in current organizational structure. Not clear who is responsible. In a specialized mental healthcare organization there are multidisciplinary meetings. It requires a different organization of care delivery and funding |
| Acceptability to stakeholders | Positive:   - Acceptable in case of experienced General Practice assistants - Good social network is a prerequisite - In cases where patients are relatively stable - Preferred over medication only; some qualified General Practice assistants are experienced and can take into account social contexts and patients’ interpersonal relations - Suitable in patients that do not benefit from CBT because they are too depressed - For the first couple of weeks where patients need to get through side effects of antidepressant medication it would be beneficial   Issues:   - Acceptable in milder depressions - In severe depression, combination therapy is preferred to enhance behavioral change and lifestyle adjustments - Not desirable in severe depression; it requires specialized care - Severe depression is often accompanied by comorbidities and increased risk of decompensation, which is not suitable for General Practice assistants |
| Other effects not captured in modelling | Not mentioned |

**Intervention substitution pair 9: Stakeholders’ perspectives on investment in combination therapy and disinvestment in individual cognitive behavioral therapy for severe depression**

| Level of evidence | No issues mentioned |
| --- | --- |
| Equity and equality considerations | Potential causes of increased inequalities/inequities are:   - Seeing a psychiatrist can be seen as threatening by patients due to stigma. It requires good coordination in practice - CBT is not suitable for everyone, a certain level of cognitive functioning, insights, and self-reflection is needed   Potential causes of decreased inequalities/inequities are:   - Suitable for the majority of adults |
| Feasibility of implementation | Positive:   - Combination therapy is current practice, no issues anticipated - Keep training up to date, which is not different from current practice   Issues:   - The availability of psychiatrists or other personnel allowed to provide the pharmacological intervention. |
| Acceptability to stakeholders | Positive:   - Patients have experienced great benefits of combination therapy - Acceptable and preferred and current practice; CBT works well if patients are not severely depressed. If that is the case, medication is important. Eventually CBT is important. Check with patients what is most beneficial - In severe depression medication is almost always necessary - Long-term effects are expected to be better in combination therapy   Neutral:   - For some patients, only CBT is sufficient; in the beginning medication is often needed   Issues:   - A minority of patients are willing to take medication due to side effects |
| Other effects not captured in modelling | Positive:   - Clinicians have more control over treatment   Negative:   - Not mentioned |

**Intervention substitution pair 10: Stakeholders’ perspectives on investment in mindfulness-based cognitive behavioral therapy and disinvestment in clinical management with maintenance medication (12 months) for prevention of recurrent depression**

| Level of evidence | - In the treatment of recurrent depressions it is proven effective, and patients with experience in mindfulness-based CBT have good experiences - Patients without experience in mindfulness-based CBT question about evidence - Mindfulness-based CBT does not seem to be implemented as frequently as medication or psychotherapy; or patients had no previous experience |
| --- | --- |
| Equity and equality considerations | Potential causes of increased inequalities/inequities are:   - Familial predisposition necessitating medication, complex comorbidities, vital depressions - Unavailability of personnel to provide the intervention   Potential causes of decreased inequalities/inequities are:   - It requires a different way of intervening; it is more about feeling and experiencing. It can serve a subgroup of patients with difficulty in cognitive functioning and recognition |
| Feasibility of implementation | Positive:   - Implementable as group therapy as it requires more mindfulness-based CBT therapists   Issues:   - It requires additional investment in personnel and training   Availability of workforce to undertake the intervention is currently unavailable |
| Acceptability to stakeholders | Positive:   - Acceptable, patients have good experiences - Clinicians’ preference if current episode is in remission - Substantial group of chronic patients in need of medication but open and willing to discontinue medication use. However, there is a need for an alternative treatment   Neutral:   - In the ideal situation provide mindfulness-based CBT and in the meantime reduce medication use or align the need for medication alongside mindfulness-based CBT   Issues:   - Clinicians’ willingness to provide the intervention, as it requires a different way of living and investment in clinicians’ own life - Dependent on the number of and time between past recurrences and its severity. In chronic depression, it is difficult to discontinue medication use - It requires self-discipline to practice in patients’ own environments - Patients’ (lack of) openness to this kind of intervention - Some people respond well to medication (biological depression) or in some cases it is desirable to maintain medication for 12 months - Patients cannot stop directly, there is a need for tapering antidepressant use or patients might be unsuccessful in discontinuation of medication due to recurrences - Patients that do not experience side effects are more reluctant to discontinue medication - Clinicians’ fear of recurrences in patients with severe depression |
| Other effects not captured in modelling | Positive:   - In the long-run, patients acquire effective tools and techniques to prevent recurrent depressions - No side-effects of medication, and in the long-run better for one’s health - In today’s digital world, mindfulness can help bring one's attention to experiences occurring in the present moment - Reflecting patients’ desire to try to quit medication use   Negative:   - Patients fear relapse and are reluctant to discontinue medication - Dependent on patient’s social network and patient’s environment |

**Intervention substitution pair 11: Stakeholders’ perspectives on investment in mindfulness-based cognitive behavioral therapy and disinvestment in preventive cognitive (behavioral) therapy for prevention of recurrent depression**

| Level of evidence | - Both interventions are effective - Patients with experience in mindfulness-based CBT have good experiences - Patients without experience in mindfulness-based CBT question about evidence - Mindfulness-based CBT does not seem to be implemented as frequently as medication or psychotherapy; or patients had no previous experience |
| --- | --- |
| Equity and equality considerations | Potential causes of increased inequalities/inequities are:   - Unavailability of personnel to provide the intervention   Potential causes of decreased inequalities/inequities are:   - It requires a different way of intervening; it is more about feeling and experiencing. It can serve a subgroup of patients with difficulty in cognitive functioning and recognition |
| Feasibility of implementation | Positive:   - Implementable as group therapy as it requires more mindfulness-based CBT therapists   Issues:   - It requires additional investment in personnel and training - The availability of workforce to undertake the intervention is currently unavailable |
| Acceptability to stakeholders | Positive:   - Acceptable to the majority of patients - Some patients with underlying persisting factors or comorbidities might benefit more of one than the other intervention - Acceptable, patients have good experiences   Issues:   - Clinicians’ willingness to provide the intervention as it requires a different way of living and investment in clinicians’ own life - It requires self-discipline to practice in patients’ own environment - Patients’ (lack of) openness to mindfulness-based CBT |
| Other effects not captured in modelling | Positive:   - Most patients who experienced recurrent depressions have already gone through several CBT trajectories; more of the same might not be effective. Mindfulness-based CBT is something different   Negative:   - Dependent on patient’s social network, and patient’s environment |

**Intervention substitution pair 12 and 13: Stakeholders’ perspectives on investment in interpersonal psychotherapy (IPT) / preventive cognitive (behavioral) therapy and disinvestment in clinical management with maintenance medication (12 months) for prevention of recurrent depression**

| Level of evidence | - Preventive CBT works equally well as clinical management with maintenance medication - The effects of antidepressants stops after discontinuation, preventive C(B)T has a prophylactic effect - Interpersonal Therapy (IPT) is an effective intervention |
| --- | --- |
| Equity and equality considerations | Potential causes of increased inequalities/inequities are:   - Familial predisposition in need of medication, complex comorbidities   Potential causes of decreased inequalities/inequities are:   - Severely depressed, comorbid disorders. Then IPT or preventive C(B)T helps to stay in conversation. |
| Feasibility of implementation | Positive:   - Existing interventions, and implementable. No issues anticipated   Neutral:   - Prevention program should be adjusted to the target population   Issues:   - There is a lack of professionals to provide IPT - Waiting list of CBT will become even longer than currently: resulting in higher workload for the therapists - Clinical management with maintenance medication is easier to implement |
| Acceptability to stakeholders | Positive:   - Acceptable to patients, it is not desirable to maintain medication use in long-term - Acceptable and clinicians’ preference if current episode is in remission - In the long-run, with IPT or preventive C(B)T, patients acquire effective tools and techniques to prevent recurrent depressions - Risks and side effects of medication, and medication does not solve the depression - IPT is especially beneficial in patients with (somatic) health issues - Acceptable from health insurers’ point of view - Substantial group of chronic patients in need of medication but open and willing to quit medication use and would welcome an alternative   Neutral:   - In the ideal situation provide mindfulness-based CBT and in the meantime reduce medication use or align the need for medication alongside mindfulness-based CBT   Issues:   - Patients prefer discontinuation of medication but fear recurrent depressive episodes - Some patients have benefits from medication (biological depression) or in some cases it is desirable to maintain medication for 12 months for example in severe depression - Patients cannot stop medication directly, there is a need for tapering antidepressant use - Patients that do not experience side effects are more reluctant to discontinue medication use - Dependent on the number of and time between past recurrences and its severity. In chronic depressions, psychotherapy alone is not desirable if discontinuing medication was unsuccessful - Some patients do not benefit from psychotherapies if they are too severely depressed or have comorbidities. Medication is helpful then, especially in the beginning - Some types of medication are hard to quit due to side effects and risk of recurrence |
| Other effects not captured in modelling | Positive:   - In the long-run; patients acquire effective tools and techniques to prevent future depressions - It enhances patient empowerment   Negative:   - Patients fear discontinuation of medication - It is dependent on patient’s social network - When patients are in remission, they sometimes find it hard to commit to therapy, some patients already had loads of CBT or IPT sessions |
